# Adult-onset Still’s disease: Switch to atypical skin manifestations under anakinra therapy

**DOI:** 10.1016/j.jdcr.2023.09.001

**Published:** 2023-09-15

**Authors:** Henning Klapproth, Birte Stroucken, Doris Helbig, Iliana Tantcheva-Poór, Mario Fabri

**Affiliations:** a Department of Dermatology and Venereology, Faculty of Medicine, University of Cologne, University Hospital of Cologne, Cologne, Germany; b Center for Molecular Medicine Cologne (CMMC), Faculty of Medicine, University of Cologne, Cologne, Germany

**Keywords:** adult onset still's disease, autoimmunity, autoinflammation, doxycycline, flagellate dermatitis, prurigo pigmentosa, skin rash

## Introduction

Adult-Onset Still’s Disease (AOSD) is a rare, autoinflammatory disease of unknown etiology, characterized by recurrent episodes of fever, arthralgia and a nonpruritic skin rash.^1^ In most cases, the rash represents as an evanescent, salmon-colored maculopapular eruption favoring sites of pressure, and coinciding with fever spikes.^1^ In contrast, prurigo pigmentosa-, flagellate-dermatitis- and dermatomyositis-like skin manifestations constitute uncommon skin findings in AOSD.^2^ Given a key role of interleukin (IL)-1 in the pathogenesis of AOSD, IL-1-receptor antagonists have been established as an effective treatment.^3^ We report an AOSD patient who experienced a paradoxical switch from common to uncommon skin manifestations after initiation of anakinra therapy. Moreover, we discuss therapeutic options for these atypical cutaneous manifestations in AOSD.

## Case report

**Fig 1:**
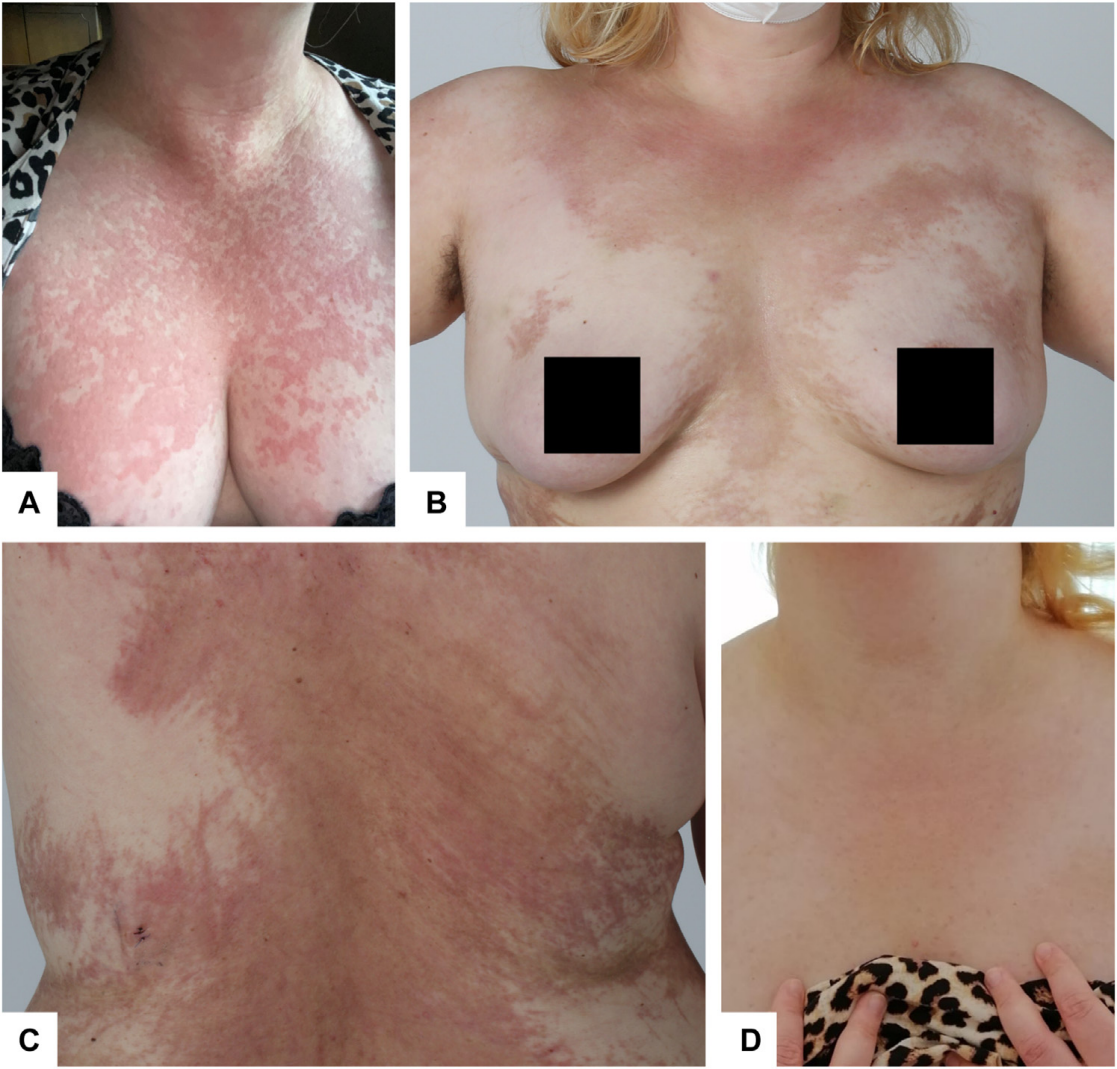
AOSD presentation with atypical skin changes. **A,** Classical skin manifestations at the time of first diagnosis. **B,** and **C,** Fixed, atypical skin manifestations resembling flagellate-dermatitis and prurigo pigmentosa-like lesions 1 month after initiation of anakinra. **D,***Restitutio ad integrum* 1 month after initiation of doxycycline and reinitiation of anakinra.

A previously healthy 29-year-old woman presented to an external hospital with an urticaria-like, salmon-colored rash that had developed over the past 2.5 months (Fig 1, *A*). The patient complained of undulant episodes of fever, a sore throat, and carpal arthralgia.

Infectious and neoplastic causes of fever were ruled out based on cultures of blood and urine as well as computed tomography -imaging of chest and abdomen and lactate dehydrogenase determination. computed tomography -radiographic finding of a retroperitoneal mass was identified by biopsy as benign adipose tissue. Laboratory results are shown in Table I. Joint sonography revealed bilateral joint effusions of carpal joints, metacarpophalangeal, proximal interphalangeal, and distal interphalangeal joint as well as both knees. In line with the Yamaguchi Criteria and the Fautrel Criteria, the diagnosis of AOSD was established. Therapy with systemic glucocorticosteroids and anakinra (IL-1–receptor antagonist) led to prompt, substantial clinical, and laboratory improvement of AOSD symptoms.

**Table I:** Initial laboratory results in the emergency department

Laboratory findings (normal range, units)	Patient results
Leukocyte count (4.0-10.0 × 10^-9^/l)	15.7 (+)
Neutrophiles (2.0-6.4 × 10^-9^/l)	13.7 (+)
Lymphocytes (1.1-3.3 × 10^-9^/l)	1.0 (−)
Monocytes (0.29-0.97 × 10^-9^/l)	0.79
Eosinophiles (0.02-0.44 × 10^-9^/l)	0.11
Basophiles (0.01-0.07 × 10^-9^/l)	0.03
Hemoglobin (12-16 g/dl)	11.5 (−)
Platelet count (150-400 × 10^-9^/l)	287
Creatinine (0.5-1.1 mg/dl)	0.65
LDH (135-214 U/l)	193
AST (< 32 U/l)	80 (+)
ALT (< 33 U/l)	124 (+)
CRP (< 0,5 mg × 10^-1^/l)	15.3 (+)
Ferritin (13-150 ng × 10^-3^/l)	24435 (+)
C3 complement (90-180 mg/dl)	202 (+)
C4 complement (10-40 mg/dl)	57 (+)
RF (<14 IU/ml)	21 (+)
CCP-Ab (<17.0 U/ml)	<10.0
ANA titer (to 1:100)	1:100 ENA negative Myositis-associated antibodies negative
Serum electrophoresis	Normal pattern, no monoclonal gammopathy.
s-IL2-receptor (158-623 U/ml)	1709 (+)

*ALT*, Alanine transaminase; *ANA*, antinuclear antibody*; AST*, aspartate aminotransferase; *CCP-ab*, cyclic citullinated pepetide antibodies; *CRP*, C-reactive protein; *ENA*, extractable nuclear antigen; *LDH*, lactate dehydrogenase; *RF*, rheumatoid factor.

**Fig 2:**
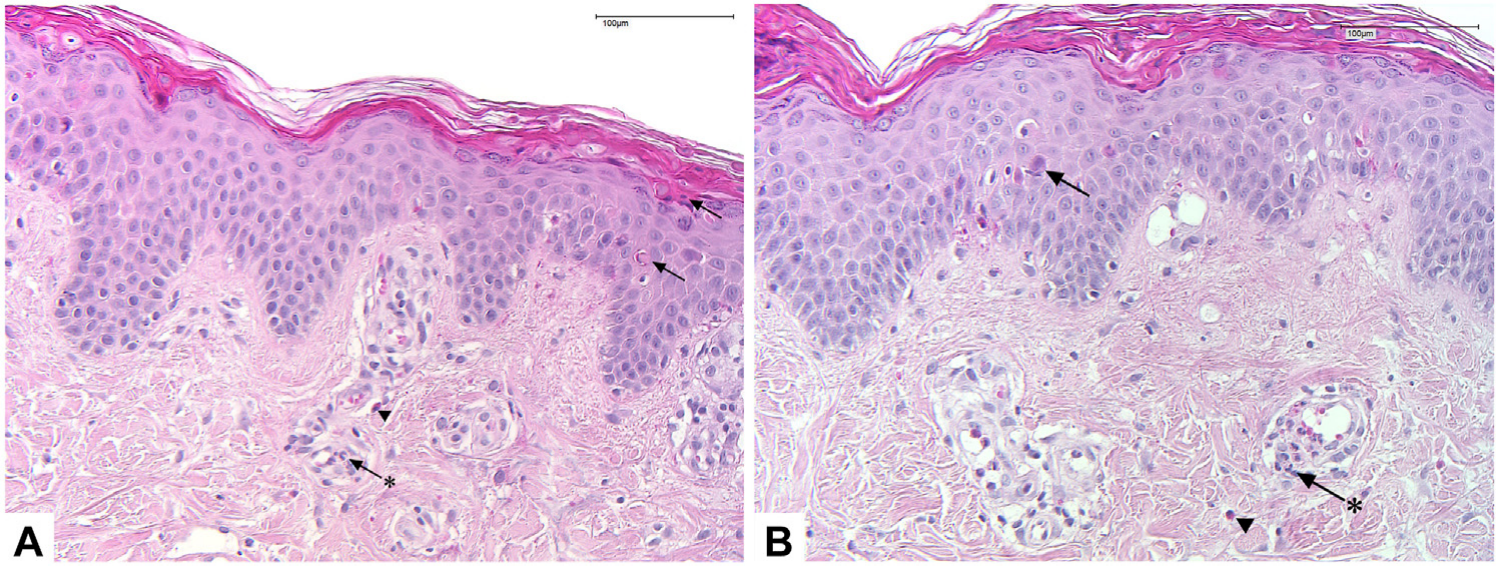
Punch biopsy with necrotic keratinocytes (*arrows*) and superficial perivascular and interstitial lymphocytic infiltration with sparse neutrophilic (*arrow* with asterisk) and eosinophilic granulocytes (*arrowhead*) (Hematoxylin and eosin stain; (**A**): magnification 10×, scale bar 100 μm; (**B**): magnification 20×, scale bar 100 μm).

One month after initiation of anakinra, the patient presented to our department with newly developed pruritic skin changes. On examination, the patient showed persisting reddish-brown, sharply demarcated macules, papules and hyperpigmented streaks, predominantly on the trunk, and proximal extremities (Fig 1, *B* and *C*). A skin punch biopsy demonstrated numerous necrotic keratinocytes and a sparse superficial infiltrate with few neutrophils as well as eosinophils (Fig 2). The clinical and histological findings prompted us to diagnose flagellate-dermatitis-like dermatitis of AOSD. However, on the flanks erythematous papules within sharply demarcated, reticulated hyperpigmentation resembling prurigo-pigmentosa-like lesions were likewise present (Fig 1, *C*). Of note, the patient had not received bleomycin and denied intake of Shiitake mushrooms.

Given that IL-6, on one hand, plays a central role in the pathogenesis of AOSD,^4^^,^^5^ and, on the other hand, seems to be involved in eruption of atypical, prurigo pigmentosa-like skin changes,^6^^,^^7^ and therapy with tocilizumab (IL-6-receptor antagonist) was initiated. Meanwhile, anakinra therapy was stopped. Within 48 hours of tocilizumab therapy and anakinra withdrawal, the patient experienced recurrence of malaise and arthralgia. After 5 days of tocilizumab and absence of clinical improvement, tocilizumab was discontinued and anakinra therapy was reinitiated, with prompt relief of systemic symptoms. However, atypical skin lesions persisted and were refractory to topical glucocorticosteroids. Given reported anti-inflammatory effects of doxycycline in this setting,^6^^,^^8^ doxycycline (100 mg BID) was added to the therapeutic regimen. Under combined anakinra and doxycycline therapy, malaise and arthralgia remained absent, and the pruritic, atypical cutaneous lesions resolved. On week-8 follow up after (re)initiation of anakinra and doxycycline, the patient continuously showed no systemic or cutaneous symptoms of AOSD (Fig 1, *D*).

## Discussion

Besides a typical evanescent, salmon-colored rash, AOSD can display other rather atypical cutaneous findings resembling flagellate dermatitis, prurigo pigmentosa, and dermatomyositis.^2^ We present a rare case of a switch from a typical to an atypical, predominantly flagellate dermatitis-like rash in an anakinra-treated AOSD patient.

An observational study providing an overview on histopathological findings of persistent papules and plaques in AOSD patients showed the presence of necrotic keratinocytes in the upper epidermis and lymphocyte-/neutrophil-dominated perivascular to interstitial infiltration of the dermis.^2^ In addition, flagellate dermatitis in the context of AOSD is histologically characterized by necrotic keratinocytes and dermal neutrophilic infiltration.^9^ Prurigo pigmentosa, first described by Nagashima et al and histologically characterized by Böer et al,^10^^,^^11^ can present with similar findings: early lesions show dense infiltrates with numerous neutrophils around the superficial plexus. Shortly thereafter, necrotic keratinocytes along with spongiosis arise. Finally, dermal infiltrates are dominated by varying amounts of lymphocytes, eosinophils, and neutrophils.

Systemic AOSD is characterized by abnormal innate immune response leading to cytokine overabundance, predominantly IL-1β and IL-18.^4^ In the case of new-onset AOSD, IL-1 receptor blockade by anakinra is commonly highly effective, as also experienced by this patient.^3^ To the best of our knowledge, hyperpigmented, reticular, or linear exanthema have not been reported as a side effect in patients receiving IL-1 receptor blockade.

Downstream effector cytokines of IL-1β such as IL-6 have been identified as drivers of both typical and atypical cutaneous inflammatory processes in AOSD.^4^^,^^5^^,^^7^ Interestingly, increased serum levels of IL-6 positively correlate with the occurrence of AOSD-typical evanescent exanthema.^5^ Although atypical skin manifestations can often present at onset of AOSD or shortly thereafter, they do not seem related to the severity of systemic AOSD symptoms.^2^ Its reported effectiveness in AOSD and possibly atypical skin changes made us switch therapy from anakinra to IL-6 blockade by tocilizumab. However, due to insufficient response, tocilizumab was discontinued. Relapses of AOSD can occur within days of withdrawal of anakinra.^4^ Consequently, anakinra was reinitiated successfully.

Tetracyclines, such as doxycycline, are known to suppress inflammatory responses in the skin by incompletely understood mechanisms without exerting substantial systemic immunosuppressive effects.^7^^,^^10^ In settings like the case presented here, doxycycline might be an effective and comparatively safe-to-use treatment option. Further research is needed to address the pathogenesis of atypical skin manifestations in AOSD in order to offer best suitable therapies.

## Conflicts of interest

Henning Klapproth, Birte Stroucken, Doris Helbig and Iliana Tantcheva-Poór declare no conflict of interest. Mario Fabri: Lecture fees: AbbVie, medupdate, Consilium (InfectoPharm), Forum für Medizinische Fortbildung; Consulting fees: Novartis, LEO Pharma; Member of professional societies/committees: Arbeitsgemeinschaft Dermatologische Forschung (Working Group for Dermatological Research), Deutsche Dermatologische Gesellschaft (German Dermatological Society), European Society for Dermatological Research, Arbeitsgemeinschaft für Dermatologische Infektiologie und Tropendermatologie (Working Group for Dermatological Infectiology and Tropical Dermatology), Deutsche STI Gesellschaft (German STI Society), Kommission Antiinfektiva, Resistenz und Therapie am Robert-Koch-Institut (Committee for Anti-Infective Agents, Resistance, and Treatment at the Robert Koch Institute, Berlin).
